# Burning mouth syndrome in younger populations: Gender disparities and bipolar tendencies

**DOI:** 10.1002/pcn5.70110

**Published:** 2025-05-15

**Authors:** Takayuki Suga, Trang Thi Huyen Tu, Motoko Watanabe, Takeshi Inoue, Akira Toyofuku

**Affiliations:** ^1^ Department of Psychosomatic Dentistry, Graduate School of Medical and Dental Sciences Institute of Science Tokyo Tokyo Japan; ^2^ Department of Basic Dental Sciences, Faculty of Dentistry University of Medicine and Pharmacy at Ho Chi Minh City Ho Chi Minh City Vietnam; ^3^ Department of Psychiatry Tokyo Medical University Tokyo Japan

Burning mouth syndrome (BMS) is a chronic condition characterized by a burning sensation or discomfort in the oral cavity, without visible lesions and radiographic changes. Its multifactorial pathophysiology has a prevalence ranging from 0.7% to 4.6%, reaching 18% in postmenopausal women.^1^ Due to the interplay of aging, neuropathic pain, and psychiatric stressors, a uniform treatment approach is challenging. Tricyclic antidepressants, particularly low‐dose amitriptyline, are the first‐line therapy for BMS‐related pain. However, stratifying patients by age and psychiatric profile may allow more targeted therapy. We therefore investigated BMS symptoms in three groups: young adults, middle‐aged adults, and older adults.

We retrospectively analyzed data from 551 patients solely diagnosed with BMS at Tokyo Medical and Dental University Hospital from April 2021 to September 2024. Patients with psychiatric histories were required to provide a referral letter from their psychiatrists. We assessed various psychiatric characteristics through questionnaires and interviews, employing the Manic Episode Screening (MES) questionnaire to identify current or past (hypo)manic episodes.^2^ The MES derives from the eight DSM‐IV‐TR criteria for mania/hypomania, with eight yes/no items. It begins with persistently elevated, expansive, or irritable mood lasting several days, followed by items on inflated self‐esteem, decreased need for sleep, increased talkativeness, racing thoughts, distractibility, increased goal‐directed activity, and risky pleasurable pursuits.

For analysis, participants were divided into three groups: young adults (<40 years, *n* = 34), middle‐aged adults (40–64 years, *n* = 250), and older adults (≥65 years, *n* = 267). Notably, young adults (32.35% male) accounted for 11 males, which is a higher proportion compared to other groups and previous BMS reports.^3^ In our earlier report, 1.1% of patients solely diagnosed with BMS had bipolar disorder; in this dataset, however, this proportion was higher at 3.09% (Table 1).^3^ The young adults demonstrated an 8.82% prevalence and the middle‐aged adults had 4.00%. Compared with a US lifetime prevalence of 2.4%, these findings suggest a notably higher proportion in younger BMS patients.^4^

**Table 1:** Diagnosis of bipolar disorder.

		Age (years)			Fisher's exact test
All participants		<40	40–64	≥65	
Diagnosis of bipolar disorder	Present	3 (8.82%)	10 (4.00%)	4 (1.50%)	0.034*
	Absent	31 (91.18%)	240 (96.00%)	263 (98.50%)	
	Total	34	250	267	551
Male					
Diagnosis of bipolar disorder	Present	2 (18.18%)	4 (11.43%)	1 (3.23%)	0.2857
	Absent	9 (81.82%)	31 (88.57%)	30 (96.77%)	
	Total	11	35	31	77
Female					
Diagnosis of bipolar disorder	Present	1 (4.35%)	6 (2.79%)	3 (1.27%)	0.3998
	Absent	22 (95.65%)	209 (97.21%)	233 (98.73%)	
	Total	23	215	236	474

*Note*: Highly significant gender difference (Fisher's exact test, P < 0.001).

When we conducted subgroup analyses by sex, males showed a higher bipolar disorder rate across all age groups. However, no significant differences emerged across the age groups within each sex, possibly due to the small sample size. Thus, sex differences in prevalence were inconclusive.

A *χ* ^2^ test among the three groups identified significant differences in five of the eight MES items (2, 4, 6, 7, and 8). A residual analysis also indicated a stronger tendency toward “soft bipolarity” in younger age groups. Odds ratios, 95% confidence intervals, and exact P‐values for these comparisons are provided in Table S1. Given this elevated tendency to soft bipolarity in younger and middle‐aged adults, there may be a risk of a manic switch when treating oral symptoms with antidepressants. Although tricyclic antidepressants pose a higher risk of inducing mania than Selective Serotonin Reuptake Inhibitors, the risk associated with low‐dose tricyclics remains unclear.^5^ If the severity of the oral symptoms necessitates the use of tricyclic antidepressants, careful monitoring at each examination is essential to detect early signs of mood elevation or manic switch. While results are not consistent regarding whether the risk of manic switch increases with escalating antidepressant doses, some studies have reported such a trend,^6^ therefore when a patient demonstrates bipolar tendencies and appears unresponsive to amitriptyline, it may be advisable not to increase the dosage. If signs of mania become evident, an early psychiatric consultation is warranted.

Furthermore, these findings suggest that awareness and screening for bipolar tendencies in BMS patients, particularly younger individuals, could facilitate earlier detection and prevention of manic episodes. Identifying subthreshold signs of bipolar disorder in this population may allow for timely referral to psychiatric care, more personalized treatment plans, and ultimately better outcomes.

From these findings, it became clear that younger patients exhibit a higher proportion of males and a higher rate of bipolar disorder, distinguishing them from the conventional characteristics of BMS. Future research will focus on differences in the symptoms of BMS specifically among young adults.

However, this study has a limitation that warrants consideration. The relatively small number of younger patients (*n* = 34) may limit the statistical power and generalizability of our findings for this subgroup. Although the overall sample size of 551 BMS patients was sufficiently large, interpretations specific to younger individuals should be made with caution. Future multicenter studies or those involving larger cohorts of young BMS patients are needed to validate these preliminary results and further elucidate the age‐specific characteristics of BMS.

## AUTHOR CONTRIBUTIONS

Takayuki Suga was responsible for data curation, formal analysis, and drafting the original manuscript. Trang Thi Huyen Tu, Motoko Watanabe, and Takeshi Inoue contributed to writing—review and editing. Akira Toyofuku was responsible for conceptualization, funding acquisition, supervision, and writing—review and editing.

## CONFLICT OF INTEREST STATEMENT

Takeshi Inoue has received personal compensation from Mochida Pharmaceutical, Takeda Pharmaceutical, Eli Lilly, Janssen Pharmaceutical, MSD, Taisho Toyama Pharmaceutical, Yoshitomiyakuhin, and Daiichi Sankyo; grants from Shionogi, Astellas, Tsumura, and Eisai; and grants and personal compensation from Otsuka Pharmaceutical, Sumitomo Pharma, Mitsubishi Tanabe Pharma, Kyowa Pharmaceutical Industry, Pfizer, Novartis Pharma, and Meiji Seika Pharma; and is a member of the advisory boards of Pfizer, Novartis Pharma, and Mitsubishi Tanabe Pharma. The other co‐authors have no conflicts of interest to declare.

## FUNDING INFORMATION

Japan Society for the Promotion of Science: 22K10141.

## ETHICS APPROVAL STATEMENT

This study was conducted in accordance with the Declaration of Helsinki and received approval from the Ethical Committee of Tokyo Medical and Dental University Hospital (approval number: D2013‐005‐04). Written informed consent was obtained from all participants.

## PATIENT CONSENT STATEMENT

N/A.

## CLINICAL TRIAL REGISTRATION

N/A.

## Supporting information

Supporting information.

## Data Availability

The data sets generated and analyzed during the current study are not publicly available due to privacy and confidentiality agreements but are available from the corresponding author on reasonable request.
